# An Opportunistic Screening Strategy for Gastric Cancer Based on Questionnaire and Sequential Serology: A Hospital-Based Cross-Sectional Study (SIGES)

**DOI:** 10.3390/jcm15010024

**Published:** 2025-12-19

**Authors:** Wen Xiang, Zhuo-Yu Li, Yan Huang, Xin-Zu Chen

**Affiliations:** 1 Gastric Cancer Laboratory, Gastric Cancer Center, Department of General Surgery, West China Hospital, Sichuan University, Chengdu 610041, China; 2 Department of Thyroid and Breast Surgery, Chengdu Fifth People’s Hospital (The Second Clinical Medical College, Affiliated Fifth People’s Hospital of Chengdu University of Traditional Chinese Medicine), Chengdu 611137, China; 3 Health Management Center, General Practice Medical Center, West China Hospital, Sichuan University, Chengdu 610041, China; 4 Ya’an Cancer Prevention and Control Center, Ya’an People’s Hospital—West China Ya’an Hospital, Sichuan University, Ya’an 625000, China; 5 Ya’an Key Laboratory for High Altitude Medicine, Ya’an People’s Hospital—West China Ya’an Hospital, Sichuan University, Ya’an 625000, China

**Keywords:** gastric cancer, screening, endoscopy, serology, CEA, CA19-9

## Abstract

**Objectives:** In the absence of massive screening programs, it is imperative to develop and validate a candidate selection strategy for opportunistic endoscopic screening (OES) targeting the early detection of gastric cancer. **Methods:** A hospital-based cross-sectional study was conducted, enrolling both health check-up controls and gastric cancer patients. Data collection included two components: (1) a questionnaire including demography, self-reported comorbidities, and family history of cancers; (2) serology including hemoglobin, carcinoembryonic antigen (CEA), and carbohydrate antigen 19-9 (CA19-9). Associations between potential variables and gastric cancer risk were assessed and the predictive efficacy of these risk factors was quantified. Sequentially, risk stratification scoring systems were constructed and their cost-effectiveness profiles were evaluated. **Results:** A total of 58,218 participants were included in the analysis, among whom 619 (1.1%) were gastric cancer patients. Multivariate analyses identified male, age >40 years, family history of gastric cancer, comorbidities of upper digestive tract benign diseases (UDTBDs), anemia, and elevated serum CEA and/or CA19-9 as independent risk factors of increasing gastric cancer risk. Cost-effectiveness analysis demonstrated that individuals, especially those symptomatic, presenting any of following conditions should be recommended for OES: (1) age ≥50 years, (2) family history of gastric cancer, and/or (3) comorbid UDTBDs. Elsewise, unclear anemia and/or elevated serum CEA and/or CA19-9 presenting among males and/or persons 41–50 years of age should be considered for OES. Notably, this selection strategy achieved a detection rate comparable to that of alternative protocols while yielding superior cost-effectiveness outcomes. **Conclusions:** The integrated strategy combining questionnaire and sequential serology represents an effective and cost-effective approach to identifying high-risk candidates for gastric cancer OES. Further investigations are warranted to develop more precise and tailored screening and surveillance protocols, with the aim of optimizing both detection rates and cost-effectiveness in clinical practice.

## 1. Introduction

Gastric cancer is a prevalent malignancy worldwide, particularly in China, characterized by high incidence and also high cancer-related mortality [1,2,3]. Globally, nearly 1 million incident gastric cancers are diagnosed annually, accounting for more than 650,000 deaths [1]. According to the latest 2018 China cancer registry report, the crude incidence and mortality rates of gastric cancer were 25.41 and 18.44 per 100,000 persons, respectively, accounting for 7.4% and 10.1% among all the new cases worldwide [4]. The “Healthy China 2030” outlines were issued in 2016, with a target of increasing the nationwide 5-year cancer survival rate by a 15% increment till 2030 [5,6]. However, the overall survival outcome of gastric cancer in China remains unsatisfactory, largely attributable to the low proportion of early-stage disease (≤20%), especially at the subnational level of China [7,8,9]. The lack of nationwide implementation of massive gastric cancer screening constitutes a major barrier to improving the early detection rate of this disease [6].

Moreover, massive gastric cancer screening remains a considerable organization and finance burden on China to date [5]. A key strategy to advance gastric cancer prevention and control is to identify, screen, and surveil the high-risk subpopulation in a systematic and cost-effective manner [5]. Therefore, opportunistic endoscopic screening (OES) of gastric cancer represents a pragmatic and feasible alternative to massive screening programs in China at present [10,11]. While OES has contributed to the detection of a substantial proportion of gastric cancer cases, the majority of these cases are diagnosed at the locally advanced or metastatic stage. Notably, locally advanced gastric cancer is frequently associated with anemia, a comorbid condition that can serve as a clinical alert to trigger OES for gastric cancer detection [12,13]. The testing of conventional serum tumor biomarkers combined with hemoglobin (HGB) assays may be a more accessible and practical pre-screening tool prior to OES implementation in China. However, standardized criteria for recommending OES in gastric cancer screening are yet to be established, particularly with respect to its diagnostic accuracy, clinical efficiency, and cost-effectiveness [10]. Although our prior research has validated the utility of a multiplex pre-OES protocol incorporating serum pepsinogen-I, pepsinogen-II, gastrin-17, and *Helicobacter pylori* testing [14,15,16], this approach has not been widely adopted in routine health check-ups, especially in rural and community healthcare settings across China. Herein, we hypothesize that a combined questionnaire–serology screening protocol, including HGB, carcinoembryonic antigen (CEA), and carbohydrate antigen 19-9 (CA19-9) testing, could facilitate and optimize gastric cancer screening workflows. Accordingly, this hospital-based cross-sectional study aimed to assess the stratified risk of gastric cancer and develop evidence-based recommendation criteria for OES candidates using a combined questionnaire–serology approach.

## 2. Methods

### 2.1. Study Design

This was a high-volume, hospital-based cross-sectional study conducted as part of the Sichuan Gastric Cancer Early Detection and Screening (SIGES) project. The study was carried out between May and December 2016 at West China Hospital, Sichuan University. The study cohort included both healthy controls and gastric cancer patients, all of whom were undergoing routine management or treatment at the aforementioned institution during the study period.

### 2.2. Eligibility

For gastric cancer patients, the inclusion criteria were as follows: (1) first diagnosis coded as ICD-10 C16 (malignant neoplasm of the stomach) in the electronic medical record system; (2) age ≥18 years; and (3) newly diagnosed case confirmed by endoscopy and biopsy, regardless of TNM stage. The exclusion criteria were as follows: (1) other types of malignancies, such as lymphoma or gastrointestinal stromal tumor; (2) lack of pathological confirmation; (3) non-incident gastric cancer cases; and (4) incomplete data retrieval. For healthy controls, the inclusion criteria were as follows: (1) selected from the health check-up registry system; (2) age ≥18 years; and (3) confirmed as tumor-free by general practitioners. The exclusion criteria were as follows: (1) presence of any tumor-suspicious findings; (2) self-reported comorbidities including precancerous lesions of atrophic gastritis, intestinal metaplasia, and intraepithelial neoplasia; and (3) incomplete data retrieval.

### 2.3. Data Collection

The information was retrieved including the questionnaire part and serology part. The questionnaire part composed of demographic features including sex (female, and male) and age (≤30, 31–40, 41–50, 51–60, 61–70, and >70 years), as well as family history of cancers in first- or second-degree relatives (none, any malignancy other than gastric cancer, and gastric cancer proband), self-reported comorbidities (none, any benign situation other than upper digestive diseases, and past or present upper digestive tract benign diseases (UDTBDs) which included *Helicobacter pylori* infection, chronic gastritis, peptic ulcer, polyp, reflux, and dyspepsia, etc.). The questionnaire items were classified as the specified features above. The serology part was collected including HGB, CEA, and CA19-9. Anemia was diagnosed as none (female HGB ≥110 g/L, and male HGB ≥ 120 g/L), mild (HGB lower than cutoff, but ≥90 g/L), and moderate–severe (HGB < 90 g/L). Seropositivity of CEA and CA19-9 was identified according to the in-house cutoffs ≥5 ng/mL and ≥30 U/mL, respectively. Additionally, the serologic multiplex of tumor biomarkers was stratified as CEA−/CA19-9−, CEA−/CA19-9+, CEA+/CA19-9−, and CEA+/CA19-9+. Total direct medical cost (TDMC) of each OES protocol was calculated as the sum of the costs of endoscopy (583.00 China Yuan (CNY)) and serology (CNY 15.00, CNY 33.00, and CNY 50.00 for blood cell count, serum CEA, and serum CA19-9 tests, respectively). Indirect medical or non-medical costs were unavailable to be collected in the present study and, therefore, they were not analyzed.

### 2.4. Scoring System of Risk Stratification and Screening Strategy

Quantitative scoring systems were developed based on the optimized predictive models. The scoring systems included two types: based on questionnaire data only and integrating both questionnaire and serology data. To enhance cost-effectiveness, a two-step sequential protocol was designed for identifying candidates eligible for OES. Additionally, a one-step protocol was established as a reference control for comparison. Two-step sequential protocol suggested those candidates for OES, including high and very high risk grades in step 1 (questionnaire); then, any participants with seropositivity were suggested for OES among mild and moderate risk grades (protocol 1, P1), or none, mild and moderate risk grades (protocol 2, P2) in the following step, step 2 (serology). The one-step protocol required simultaneous serological tests for all at the moment of the questionnaire (protocol 3, P3). Only individuals stratified as high-risk or very high-risk based on the combined assessment (P3) were designated as OES candidates.

### 2.5. Ethics

The serial SIGES studies were approved by the Biomedical Ethical Committee of West China Hospital, Sichuan University (id: 2015-151-V2, 2 November 2015; 2018-215-V1, 6 August 2018). This study retrospectively collected the observations’ electronic medical records. Therefore, the informed consent was waived due to the retrospective nature, while the personal information was anonymized during the whole procedure of analyzing and reporting data.

### 2.6. Registration and Reporting

The protocol was reported elsewhere (ClinicalTrials.gov Identifier: NCT03597672). This cross-sectional study was qualified according to the Strengthening the Reporting of Observational Studies in Epidemiology (STROBE) statement [17] and the REporting of studies Conducted using Observational Routinely collected Data (RECORD) [18].

### 2.7. Statistics

Cross-sectional baselines of the observations were compared by Chi-square test for categorical variables and Wilcoxon rank-sum test for ranked variables or continuous variables. Both univariate and multivariate analyses were performed by Logistic regression, and odds ratios (ORs) or adjusted odds ratios (aORs) with 95% confidence intervals (CIs) were estimated. Bootstrap resampling and estimation was performed with 200 rounds of repetition to optimize the model. Additionally, various scoring systems were generated from the model coefficients of Logistic regression, while the point of each variable was fixed by minimal common divisor and round-off methods. Non-parametric receiver operating characteristic curve (ROC) analysis was used to assess the capability of predicting gastric cancer cases, while detection rate (DR = detected cases/all observations × %), sensitivity (SEN), specificity (SPE), correction rate (COR), positive likelihood ratio (PLR), and negative likelihood ratio (NLR) were estimated. The strength of area under ROC (AUC), SEN, SPE, COR, or DR was classified as weak (0.5–0.7), moderate (0.7–0.9), and strong (0.9–1). Additionally, the maximal Youden index (= SEN + SPE) was calculated to determine the statistical optimal cutoff of score index. The AUC with standard error was calculated. The difference in AUCs was compared by the Z test. The number needed to screen (NNS) was estimated in diverse subsets, compared to the none-risk subset. A rapid health economic assessment was performed, while cost-effectiveness ratio (CER) and incremental cost-effectiveness ratio (ICER) were calculated based on the total direct medical cost (TDMC) and DR of each protocol. In addition, false-positive rate (FPR) and false-negative rate (FNR) were calculated. Sensitivity analysis of CERs was performed by adjusting the amount of health controls from 1-fold to 10-fold among proposed protocols. Two-sided *p* value <0.05 was considered as statistical significance. The STATA/SE 14.0 software was used for statistical analysis.

## 3. Results

### 3.1. Baseline Features

**Figure 1:**
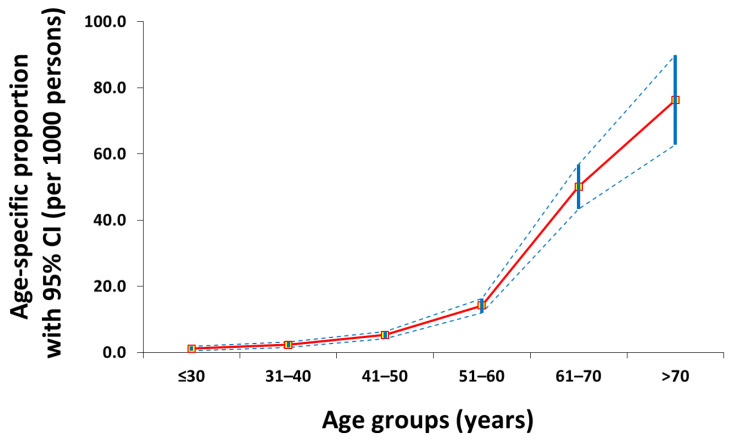
Age-specific proportion curve (red line) of gastric cancer patients with 95% confidence intervals (blue intermittent lines) in this cross-sectional study.

A total of 58,218 observations were analyzed in this cross-sectional study including 619 gastric cancer patients (1.1%) (Figure 1). Greater proportions of males (71.4%), elders (51.4%), comorbid UDTBDs (30.2%), family history of gastric cancer (6.3%), anemia (35.7%), CEA seropositivity (21.3%), and CA19-9 seropositivity (19.9%) were found in the gastric cancer group (*p* < 0.05) (Table 1).

### 3.2. Risk Factors and Scoring Systems

**Figure 2:**
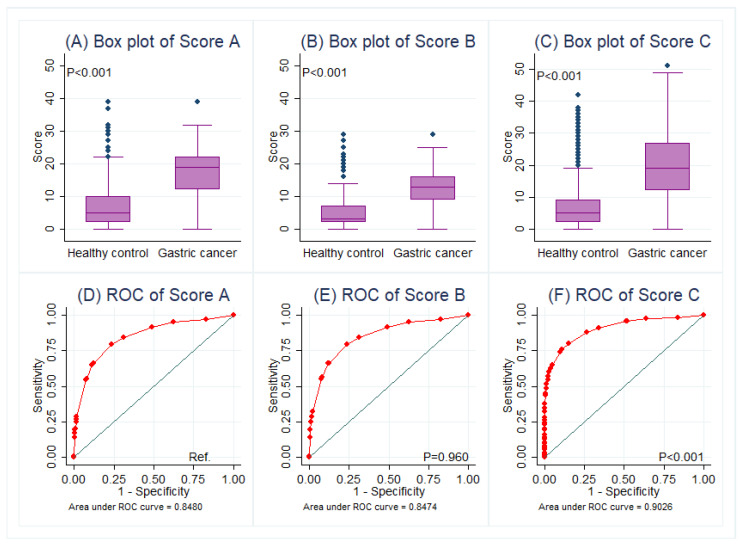
Comparisons of scoring systems in box plots (**A**–**C**) and its predictive performance between healthy controls and gastric cancer patients in ROC curves (**D**–**F**), in which the AUC of Score C was preferred. Abbreviations: Ref., reference; ROC, receiver operating characteristic curve.

Univariate analysis and four models of multivariate regression were performed (Table 2). Particularly, model 4 found that male, age >40 years, family history of gastric cancer, comorbidity of UDTBDs, anemia, and seropositivity of CEA and/or CA19-9 were independent risk factors for gastric cancer. Based on model 3 and 4, three scoring systems were established (Table 3). Scoring systems A and B were only based on the questionnaire part, while scoring system C contained both the questionnaire and serology parts. The ROC of Score C performed better to predict gastric cancer (AUC = 0.903, 95% CI 0.889–0.917) in contrast with Score A (*p* < 0.001), but the AUC of Score B was not superior to that of Score A (*p* = 0.960) (Figure 2). Serology could provide an additional benefit in predicting gastric cancer.

### 3.3. Screening Strategy and Prediction Strength

**Figure 3:**
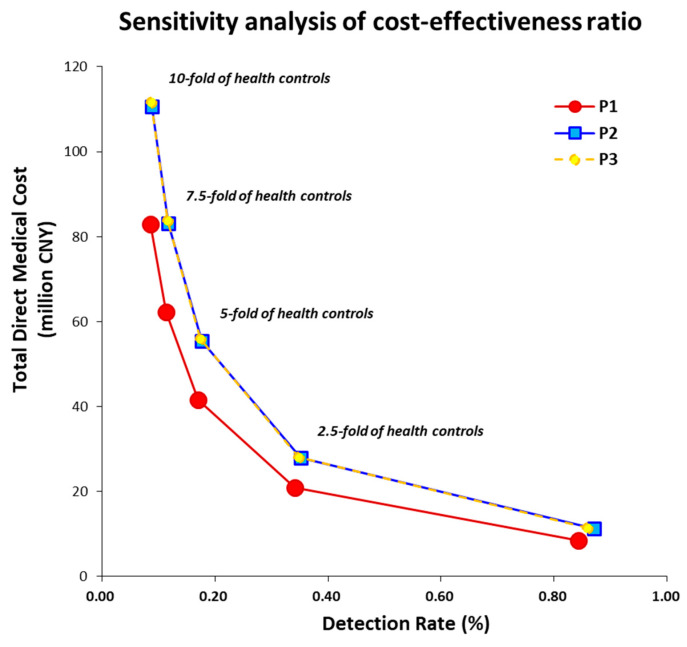
Sensitivity analysis of cost-effectiveness ratios by adjusting the amount of health controls from 1-fold to 10-fold among three proposed protocols, among which protocol 1 (P1) was preferred. Abbreviation: China Yuan, CNY.

Moderate predictive performance of selected scoring systems B and C was demonstrated according to cutoffs by maximal Youden index (Table 4). SEN and SPE were 67.2% and 87.5% for score B and 80.8% and 82.7% for score C, respectively. The performance of risk stratification based on serial cutoffs and combinations was showed by ORs and NNSs in the three protocols P1, P2, and P3 (Table 5). The DRs of protocols P1, P2, and P3 were 8.43‰, 8.71‰, and 8.59‰, respectively (Table 6). The two-step protocol P1 was preferred, while the other two-step protocol P2 was considered in willing-to-pay manner (Table 6). CER and ICER analyses indicated that the protocol P3 was dominated (Table 6). Sensitivity analysis of CERs showed the protocol P1 of OES was always preferred due to the consistently better cost-effectiveness performance (Figure 3).

## 4. Discussion

This hospital-based cross-sectional study simultaneously enrolled health check-up participants and gastric cancer patients, aiming to evaluate the efficacy of questionnaire and sequential serology strategy for identifying high-risk candidates eligible for OES of gastric cancer. Our findings indicate that individuals presenting with the following specific clinical features, particularly if symptomatic, should be directly recommended for OES: (1) age >50 years; (2) family history of gastric cancer; and/or (3) comorbid UDTBDs. Elsewise, unclear anemia and/or elevated serum CEA and/or CA19-9 presenting among males and/or persons 41–50 years of age should be considered for OES. Notably, this selection strategy achieved a detection rate comparable to that of alternative protocols while yielding superior cost-effectiveness outcomes.

English National Cancer Diagnosis Audit 2018 data analysis found that detailed associations between presenting specific symptoms and cancer sites, which could promote cancer control awareness campaigns, and diagnostic strategies post-presentation if cancer suspected [12]. Based on the Denmark and UK Biobanks, comprehensive cancer prediction models could be built through combining medical history, available clinical disease trajectories, text-mined basic health factors, and family histories for 20 major cancer types [19]. Therefore, OES for gastric cancer requires full understanding of the warning symptom and risk factors. In addition, the combination with biomarkers may strengthen the effectiveness of cancer screening protocols [20,21]. For proposed screening protocols, analyses of endoscopic uptake rate, gastric cancer detection rate, and early gastric cancer detection rate should be valuable to be accessed, which can positively impact the mortality rate of gastric cancer [22,23,24]. Without those analyses, further validation investigations are required before generalizability.

In 2024, the National Health Commission of the People’s Republic of China issued the first official guidelines on screening, early diagnosis, and early treatment of gastric cancers [25]. The identification of high-risk candidates for screening contains specified comorbidities, including *Helicobacter pylori* infection, chronic atrophic gastritis, gastric ulcer, gastric polyp, remnant stomach, Menetrier disease, and pernicious anemia, etc. [25]. Providing more severe precancerous lesions of intestinal metaplasia or intraepithelial neoplasia, intervals of surveillance should be narrowed accordingly [25]. Therefore, patients with precancerous lesions of atrophic gastritis, intestinal metaplasia, and intraepithelial neoplasia were excluded from the control group. When comparing the present finding with the aforementioned guidelines (2024 edition), supplementary suggestion can be obtained provided that self-reported UDTBDs, as well as unclear anemia and/or elevated of CEA and/or CA19-9 among specific subpopulation, could be associated with increased risk of gastric cancer. These situations evidenced from the present study can be recommended to expend the candidates of OES and obtain better DR. Moreover, these findings are consistent with the guidelines (2024 edition) that serology should not be used alone, but for primary screening before sequential OES.

Moreover, past or present upper digestive tract benign diseases (UDTBDs), including *Helicobacter pylori* infection, chronic gastritis, peptic ulcer, polyp, reflux, and dyspepsia, were combined into single parameter. In our findings, UDTBDs totally composed of 30.2% among gastric cancer patients, in contrast with 3.0% among health check-up persons (aOR = 9.73, 95% CI 7.76–12.21). UDTBDs including *Helicobacter pylori* infection, lifestyle, and dietary habits can initiate the Correa’s cascade to dynamically develop intestinal-type gastric cancer [26,27]. Therefore, the detailed inquiry of symptom and comorbidity may be meaningful and sensitive to recommend OES candidates by specialists or general practitioners. However, many patients with gastric precancerous conditions or early gastric cancer remain asymptomatic and identifying these individuals represents a primary objective of OES. The approach of self-reported symptoms or UDTBDs may partially omit patients with early diseases. The error cannot be prevented in the present study due to its retrospective nature, but our findings showed positive results of predictive association to some extent.

Family history of gastric cancer can be an independent or co-exposure risk factor for familial gastric cancer [28,29]. Co-exposure of *Helicobacter pylori* infection or unhealthy diet and lifestyle among long-term co-residents is one of the possible reasons resulting in familial gastric cancer. Meanwhile, the risk of gastric cancer and gastric adenoma appeared higher, provided both parents and siblings were diagnosed with gastric cancers [30]. Moreover, if the precancerous mucosal abnormalities are recorded in a person’s first-degree relatives, the stratification of gastric cancer risk for this particular person may be raised [31]. Among persons with *Helicobacter pylori* infection and also family history of gastric cancer in first-degree relatives, eradication treatment and maintaining its negativity could reduce those risks of gastric cancer [28,32]. In addition, hereditary diffuse gastric cancer (HDGC) is a subset of familial cancer syndromes, specifically associated with germline mutations to the E-cadherin (*CDH1*) gene [33,34]. A systematic review recommended the prophylactic total gastrectomy in *CDH1* mutation-positive persons with family history of gastric cancer [35]. Therefore, persons with family history of gastric cancer are recommended for not only the OES, but also the screening for *Helicobacter pylori* infection and *CDH1* mutation. The eradication of *Helicobacter pylori* is subsequently suggested, but prophylactic total gastrectomy should be carefully considered with caution among high-selected feasible candidates due to inadequate evidence.

In the guidelines by the National Health Commission of the People’s Republic of China and relevant collaborations, serology alone has not been recommended for screening both gastric cancer and its precancerous high-risk events as common practice [10,25]. However, serum *Helicobacter pylori* antibody, pepsinogen-I, pepsinogen-II, and gastrin-17 have been widely researched and practiced in health check-up, screening, and surveillance of gastric cancer and its precancerous high-risk events, which shows promising performance in the stratification of gastric cancer risk [15,36,37]. Conventional serum tumor biomarkers such as CA724, CEA, CA19-9, and CA242 are associated with significantly increased risks of gastric cancer and its precancerous high-risk events [38,39,40,41]. In addition, non-specific reduction in HGB is similarly associated with increased risks of gastric cancer and its precancerous high-risk events [42,43]. More novel serologic and blood tests are warranted in the field of screening and early detection of gastric cancer [44,45]. Although they have not been recommended in the guidelines yet [25], the application of their combinations remains interesting in further investigations. It was found that strategies incorporating risk stratification using alternative measurements, such as serologic tests, are more cost-effective for selecting OES candidates [46]. Moreover, the protocol of endoscopic surveillance among the high-risk subpopulation still requires further investigation regarding follow-up interval and the modification of stratification by alternative measurements [47].

It needs to be clarified that the UDTBDs were identified by self-reporting manner from electronic medical records of West China Hospital, which could systematically underestimate the prevalence of UDTBDs among health check-up individuals. Comprehensive linkage with individual’s in-house or outside medical records is not available by now in Sichuan Province. Therefore, the underestimate of UDTBDs’ prevalence might be caused by the unwillingness to self-report or the absence of endoscopy or other tests to diagnose. Indeed, our previous study found that the prevalence of *Helicobacter pylori* was decreased from 53.1% to 30.7% between 2009–2010 and 2019–2021 among health check-up individuals in West China Hospital [48]. It is consistent with the results of meta-analysis in China [49], but both are significantly higher than the self-reported prevalence. Additionally, a report documented that the prevalence of atrophic gastritis was as high as 25.8% among symptomatic patients [50] but, contrary our previous study, found that the prevalence of atrophic gastritis was just 15.9/1000 persons among the symptom-free health check-up population [15]. It is the reason why the prevalence was diverse between different observations.

There were still several limitations that needed consideration with caution. First, this was a hospital-based cross-sectional population, rather than a natural population. In the present study, the proportion of gastric cancer was up to 1.06%, which was much higher than the crude incidence rate of gastric cancer in China (25.41/100,000 persons) [4]. A certain sampling bias may be introduced, and the positive findings are possibly amplified due to the scale-up effect. In addition, a single hospital-based study may impair the generalizability of the results, rather than multicenter recruitment. Second, due to the resource-intensive nature of endoscopy, massive endoscopic screening of gastric cancer is impracticable among the general health check-up population. Opportunistic endoscopic screening is recommended among the high-risk subpopulation instead. Therefore, the health check-up did not involve endoscopic confirmation of the absence of gastric cancer in the present retrospective study. However, early gastric cancer is often asymptomatic, so the risk of case misclassification might exist theoretically. Further prospective study, including endoscopy-proven cancer-free controls, is expected for robust correction. Third, in the present study, internal cross-validation, involving training and validation settings, was not performed. Additionally, external validation is expected to be performed in the sequential cross-sectional study at West China Hospital or other hospitals. Fourth, unhealthy lifestyles and dietary habits, such as smoking, heavy drinking, high-salt diet, and pickled-food diet, were defined as risk factors for gastric cancer screening. However, because of a lot of missing data, unhealthy lifestyles and dietary habits were not included in the questionnaire of the present study. Fifth, the serologic stratification combining serum pepsinogen-I, pepsinogen-II, and gastrin-17 may provide incremental accuracy in selecting OES candidates. Finally, the gastric cancer patients at West China Hospital were composed of a small proportion of early-stage cases (<20% among surgical patients) [7,8]. Such a limited number of early gastric cancers will markedly impair the test power and increase the risk of type II error. Consequently, the present study did not perform subgroup analysis restricted to early gastric cancer only, which may exaggerate the effectiveness of early detection instead. Namely, the confirmation of the above findings should be performed carefully and requires more robust investigations.

## 5. Conclusions

Opportunistic endoscopic screening serves as a critical modality for the early detection of gastric cancer and its precancerous conditions in endemic regions lacking massive screening programs. A combined strategy of questionnaire-based risk stratification followed by sequential serological testing represents a cost-effective and efficient approach to identify high-risk individuals eligible for opportunistic endoscopic screening for gastric cancer. Further investigations into more precise, personalized screening and surveillance protocols are warranted to enhance the detection rate and optimize cost-effectiveness.

## Figures and Tables

**Table 1:** Baseline features and serologic results of healthy controls and gastric cancer patients.

Risk Factors	Healthy Controls (N = 57,599)		Gastric Cancers (N = 619)		*p* Values
	*n*	%	*n*	%	
Questionnaire part					
Sex					<0.001 *
Female	24,415	42.4	177	28.6	
Male	33,184	57.6	442	71.4	
Age (yrs)					<0.001 ^#^
≤30	8794	15.3	10	1.6	
31–40	13,486	23.4	31	5.0	
41–50	18,920	32.9	100	16.2	
51–60	11,145	19.4	160	25.9	
61–70	3886	6.8	205	33.1	
>70	1368	2.4	113	18.3	
Family history of malignancies					0.008 ^#^
None	50,805	88.2	528	85.3	
Non-gastric malignancies	5983	10.4	52	8.4	
Gastric cancer	811	1.4	39	6.3	
Comorbidity					<0.001 *
None or non-digestive	55,884	97.0	432	69.8	
UDTBDs	1715	3.0	187	30.2	
Serology part					
Anemia					<0.001 ^#^
None	56,292	97.7	398	64.3	
Mild	1162	2.0	125	20.2	
Moderate–severe	145	0.3	96	15.5	
Serum CA19-9					<0.001 *
Negative	55,850	97.0	496	80.1	
Positive	1749	3.0	123	19.9	
Serum CEA					<0.001 *
Negative	56,444	98.0	487	78.7	
Positive	1155	2.0	132	21.3	
Serologic multiplex					<0.001 ^#^
CEA–/CA19-9−	54,814	95.2	418	67.5	
CEA–/CA19-9+	1036	1.8	78	12.6	
CEA+/CA19-9−	1630	2.8	69	11.2	
CEA+/CA19-9+	119	0.2	54	8.7	

Abbreviations: CA19-9, carbohydrate antigen 19-9; CEA, carcinoembryonic antigen; UDTBDs, upper digestive tract benign diseases; yrs, years. * Chi-square test. # Wilcoxon rank-sum test.

**Table 2:** Risk factors of gastric cancer identified by Logistic regression with bootstrap resampling.

Risk Factors	Univariate	Model 1	Model 2	Model 3	Model 4
	OR (95% CI)	aOR (95% CI)	aOR (95% CI)	aOR (95% CI)	aOR (95% CI)
Sex (male vs. female)	1.84 (1.55–2.18)	1.69 (1.36–2.11)	1.69 (1.35–2.12)	1.46 (1.24–1.73)	1.69 (1.37–2.09)
Age (yrs)					
≤30	Ref.	Ref.	Ref.	Ref.	Ref.
31–40	2.02 (1.02–4.01)	1.43 (0.71–2.88)	1.43 (0.66–3.10)	Ref.	Ref.
41–50	4.65 (2.35–9.20)	2.78 (1.50–5.17)	2.78 (1.35–5.76)	2.54 (1.74–3.70)	2.16 (1.48–3.14)
51–60	12.62 (6.28–25.38)	7.74 (4.09–14.67)	7.75 (3.73–16.08)	6.42 (4.54–9.07)	6.01 (4.10–8.79)
61–70	46.39 (23.37–92.08)	22.92 (12.49–42.06)	22.93 (11.20–46.96)	26.34 (18.77–36.97)	17.50 (12.16–26.19)
>70	72.64 (36.45–144.76)	21.86 (11.45–41.75)	21.88 (10.77–44.45)		
Family history of malignancies					
None	Ref.	Ref.	Ref.	Ref.	Ref.
Non-gastric malignancies	0.84 (0.63–1.11)	1.04 (0.72–1.50)	1.04 (0.77–1.42)	Ref.	Ref.
Gastric cancer	4.63 (3.21–6.68)	5.72 (3.91–8.38)	5.73 (3.90–8.40)	4.49 (3.02–6.66)	5.71 (3.91–8.32)
Comorbidity (UDTBDs vs. none or non-digestive)	14.11 (11.63–17.10)	9.69 (7.73–12.13)	9.69 (7.90–11.88)	9.99 (8.20–12.18)	9.73 (7.76–12.21)
Anemia (vs. none)					
Mild	23.30 (18.46–29.42)	26.07 (19.54–34.78)	26.06 (19.29–35.22)	/	26.07 (19.78–34.35)
Moderate–severe	94.30 (72.28–123.03)	99.23 (67.84–145.15)	99.19 (67.58–145.60)	/	99.61 (68.49–144.89)
Serum CA19-9 (positive vs. negative)	7.92 (6.42–9.76)	3.21 (2.39–4.31)	/	/	/
Serum CEA (positive vs. negative)	13.25 (10.74–16.33)	5.41 (4.08–7.18)	/	/	/
Serologic multiplex (vs. CEA–/CA19-9−)					
CEA–/CA19-9+	5.55 (4.32–7.13)	/	3.19 (2.24–4.53)	/	3.18 (2.28–4.44)
CEA+/CA19-9−	9.87 (7.61–12.82)	/	5.38 (4.06–7.13)	/	5.38 (3.90–7.41)
CEA+/CA19-9+	59.51 (42.13–84.04)	/	17.59 (10.77–28.73)	/	17.59 (10.87–28.47)

Abbreviations: aOR, adjusted odds ratio; CA19-9, carbohydrate antigen 19-9; CEA, carcinoembryonic antigen; CI, confidence interval; Ref., reference; UDTBDs, upper digestive tract benign diseases; yrs, years.

**Table 3:** The coefficients of risk factors and alternative scoring systems to predict gastric cancer.

Risk Factors	Model 3		Model 4		
	Coefficient *	Score A	Coefficient *	Score B	Score C
Questionnaire					
Male	0.380	2	0.524	2	2
Age 41–50 yrs	0.932	5	0.769	3	3
Age 51–60 yrs	1.859	10	1.793	7	7
Age >60 yrs	3.271	17	2.862	11	11
Family history of gastric cancer	1.502	8	1.742	7	7
Comorbidity of UDTBDs	2.302	12	2.275	9	9
Serology					
Anemia (mild)	/	/	3.261	/	12
Anemia (moderate–severe)	/	/	4.601	/	18
Serologic CEA−/CA19-9+	/	/	1.158	/	4
Serologic CEA+/CA19-9−	/	/	1.682	/	6
Serologic CEA+/CA19-9+	/	/	2.867	/	11

Abbreviations: CA19-9, carbohydrate antigen 19-9; CEA, carcinoembryonic antigen; UDTBDs, upper digestive tract benign diseases; yrs, years.* Coefficients were generated from those Logistic regression models.

**Table 4:** Predictive strength of selected scoring systems B and C.

	SEN	SPE	COR	PLR	NLR
Cutoffs of Score B					
≥2	95.2%	37.6%	38.2%	1.5	0.1
≥4	84.2%	68.8%	69.0%	2.7	0.2
≥6 (by maximal Youden index)	67.2%	87.5%	87.3%	5.4	0.4
≥8	33.1%	97.4%	96.7%	12.5	0.7
Cutoffs of Score C					
≥2	97.7%	35.6%	36.3%	1.5	0.1
≥4	91.0%	65.5%	65.8%	2.6	0.1
≥6 (by maximal Youden index)	80.8%	83.7%	83.6%	5.0	0.2
≥8	66.9%	93.7%	93.4%	10.6	0.4

Abbreviations: COR, correction rate; NLR, negative likelihood rate; PLR, positive likelihood rate; SEN, sensitivity; SPE, specificity. Note: Youden index = SEN + SPE.

**Table 5:** Protocols of questionnaire and serology combination for OES candidates.

OES Protocol by Risk Stratification	Sum	Healthy Controls		Gastric Cancers		OR (95% CI)	NNS
		*n*	%	*n*	%		
Two-step sequential protocols							
Score B system (without serology)							
0 (None)	21,657	21,627	99.86	30	0.14	Ref.	Ref.
2–3 (Mild)	18,088	18,020	99.62	68	0.38	2.72 (1.77–4.18)	422
4–5 (Moderate)	10,845	10,740	99.03	105	0.97	7.05 (4.69–10.58)	121
6–7 (High, for OES)	5898	5687	96.42	211	3.58	26.75 (18.23–39.25)	30
≥8 (Very high, for OES)	1730	1525	88.15	205	11.85	96.91 (65.83–142.65)	9
P1: Mild and moderate risk grades (Score B: 2–5 for serology)							
Any seronegative	26,730	26,632	99.63	98	0.37	Ref.	Ref.
Any seropositive (for OES)	2203	2128	96.60	75	3.40	9.58 (7.07–12.98)	33
P2: None, mild and moderate risk grades (Score B: 0–5 for serology)							
Any seronegative	47,250	47,138	99.76	112	0.24	Ref.	Ref.
Any seropositive (for OES)	3340	3249	97.28	91	2.72	11.79 (8.92–15.58)	41
One-step protocol							
P3: Score C system (all for serology)							
0 (None)	20,520	20,506	99.93	14	0.07	Ref.	Ref.
2–3 (Mild)	17,269	17,227	99.76	42	0.24	3.57 (1.95–6.54)	572
4–5 (Moderate)	10,525	10,462	99.40	63	0.60	8.82 (4.94–15.75)	189
6–7 (High, for OES)	5849	5763	98.53	86	1.47	21.86 (12.42–38.48)	72
≥8 (Very high, for OES)	4055	3641	89.79	414	10.21	166.55 (97.66–284.02)	10

Abbreviations: CI, confidence interval; NNS, number needed to screen; OR, odds ratio; OES, opportunistic endoscopic screening; Ref., reference.

**Table 6:** Cost-effectiveness analysis of OES protocols.

OES Protocol	DR (‰)	FPR (%)	FNR (%)	TDMC (Million CNY)	CER	ICER	Recommendation
P1	8.43	16.2	20.7	8.57	10.16	Ref.	Preferred
P2	8.71	18.2	18.1	11.35	13.04	101.35	Willing-to-pay
P3	8.59	16.3	19.2	11.48	13.37	188.40	Dominated

Abbreviations: CER, cost-effectiveness ratio; CNY, China Yuan; DR, detection rate; FNR, false-negative rate; FPR, false-positive rate; ICER, incremental cost-effectiveness ratio; OES, opportunistic endoscopic screening; Ref., reference; TDMC, total direct medical cost.

## Data Availability

The in-house data will be available by emailing the corresponding author chenxinzu@scu.edu.cn.
